# Sigh and Eupnea Rhythmogenesis Involve Distinct Interconnected Subpopulations: A Combined Computational and Experimental Study^1^,^2^,^3^

**DOI:** 10.1523/ENEURO.0074-14.2015

**Published:** 2015-04-22

**Authors:** Natalia Toporikova, Marc Chevalier, Muriel Thoby-Brisson

**Affiliations:** 1 Department of Biology, Washington and Lee University, Lexington, Virginia 24450; 2 Institut de Neurosciences Cognitives et Intégratives d’Aquitaine, CNRS UMR 5287, Université de Bordeaux, 33076 Bordeaux, France

**Keywords:** computational model, in vitro, pre-Bötzinger complex, respiratory network, sigh generation

## Abstract

Neural networks control complex motor outputs by generating several rhythmic neuronal activities, often with different time scales. One example of such a network is the pre-Bötzinger complex respiratory network (preBötC) that can simultaneously generate fast, small-amplitude, monophasic eupneic breaths together with slow, high-amplitude, biphasic augmented breaths (sighs). However, the underlying rhythmogenic mechanisms for this bimodal discharge pattern remain unclear, leaving two possible explanations: the existence of either reconfiguring processes within the same network or two distinct subnetworks. Based on recent *in vitro* data obtained in the mouse embryo, we have built a computational model consisting of two compartments, interconnected through appropriate synapses. One compartment generates sighs and the other produces eupneic bursts. The model reproduces basic features of simultaneous sigh and eupnea generation (two types of bursts differing in terms of shape, amplitude, and frequency of occurrence) and mimics the effect of blocking glycinergic synapses. Furthermore, we used this model to make predictions that were subsequently tested on the isolated preBötC in mouse brainstem slice preparations. Through a combination of *in vitro* and *in silico* approaches we find that (1) sigh events are less sensitive to network excitability than eupneic activity, (2) calcium-dependent mechanisms and the *I_h_* current play a prominent role in sigh generation, and (3) specific parameters of *I_h_* activation set the low sensitivity to excitability in the sigh neuronal subset. Altogether, our results strongly support the hypothesis that distinct subpopulations within the preBötC network are responsible for sigh and eupnea rhythmogenesis.

## Abstract (precis)

How a single neural network can generate several rhythmic activities at different time scales remains an open question. Here, in addition to the already described reconfiguring process, we propose a new mechanism by which the respiratory network can generate simultaneously two distinct inspiration-related activities (eupnea and sigh) at different frequencies.

## Significance Statement

How a single neural network can generate several rhythmic activities at different time scales remains an open question. Here, in addition to the already described reconfiguring process, we propose a new mechanism by which the respiratory network can generate simultaneously two distinct inspiration-related activities (eupnea and sigh) at different frequencies. By combining physiological recordings of fictive inspiratory activities *in vitro* with computational modeling, we test the possibility that eupnea and sigh rhythmogenesis rely on the interaction between two distinct subpopulations within the respiratory network. Our *in silico* and *in vitro* results provide evidence supporting this hypothesis and thus bring new insights regarding possible mechanisms allowing one network to generate rhythmic activities differing in terms of frequencies of occurrence.

## Introduction

In several motor systems, the generation of multiple rhythms by a single central network is often attributed to circuit reconfiguring processes. For example, in the crustacean stomatogastric nervous system, the two interacting pyloric and gastric networks under certain neuromodulatory conditions can generate distinctive motor patterns through functional rearrangements of these circuits (Dickinson et al., 1990; Meyrand et al., 1991; Blitz and Nusbaum, 1997; Combes et al., 1999; Faumont et al., 2005). A similar reconfiguration process has also been described in the mouse respiratory network of the pre-Bötzinger complex (preBötC) for gasping motor activity produced under anoxic conditions. Indeed, a decrease in synaptic inhibition enables all post-inspiratory and expiratory neurons (interneurons with functionality related to other phases of the respiratory cycle) to become active during inspiration (Lieske et al., 2000; Paton et al., 2006). Interestingly, the respiratory neural network is also able to generate a third type of respiration-related activity, namely sighs (or augmented breaths). Sighs are biphasic large-amplitude inspiratory events generated intermittently with eupneic activity but at a lower frequency and higher amplitude (Cherniack et al., 1981; Orem and Trotter, 1993; Takeda and Matsumoto, 1997; Lieske et al., 2000; Tryba et al., 2008; Koch et al., 2013; Chapuis et al., 2014). The shape of sigh motor events depends on inhibitory connections among inspiratory neurons, since disturbances of glycinergic inhibition convert sigh bursts from biphasic to monophasic (Lieske et al., 2000; Chapuis et al., 2014).

In contrast to gasps, the generation of sigh activity appears not to derive from a network reconfiguring process. Indeed, the vast majority of the neurons active during eupnea are also active during sighs and no functional remodeling within the network has been identified so far. Intriguingly, two previous *in vitro* studies have described a very small subpopulation of inspiratory neurons that are active exclusively during sigh-like activity (Tryba et al., 2008; Chapuis et al., 2014). However, the specific role of these cells in the production of sigh bursts remains unclear, largely because the so called sigh-only cells represent a very limited neuronal population (the existing recordings suggest that these neurons constitute less than 5% of the inspiratory neurons of the respiratory network), and thereby are difficult to target for investigation. Furthermore, the excitatory glutamatergic connections are widespread within the respiratory network and likely to be involved in the production of eupneic and sigh bursts. Such a reciprocally and intensively connected network makes distinguishing between sigh and eupnea using classical electrophysiological approaches problematic. Consequently, whether two distinct subpopulations within the respiratory network are responsible for the generation of sigh and eupnea remains an open question. Here, we addressed this issue by combining computational modeling and *in vitro* experimentation. We designed a two-compartment computational model for sigh and eupnea subpopulations of neurons with several different parameters reflecting distinct burst-generating mechanisms. The sigh subpopulation generates low-frequency rhythm based on slow intracellular Ca^2+^ oscillations and the eupnea subnetwork generates fast oscillations mainly driven by activation/inactivation of the persistent Na^+^ current. We also tested connections between these two subpopulations and found that a model in which eupnea compartment inhibits sigh compartment and sigh compartment excites eupnea compartment is consistent with experimental data obtained *in vitro*. Furthermore, we used this model to make several predictions that we tested using *in vitro* electrophysiological recordings of fictive inspiration-related activities generated in brainstem slices.

## Materials and Methods

### Computational model

The sigh−eupnea computational network combines two single-compartment models representing sigh and eupnea subpopulations, connected through excitatory and inhibitory synapses. We assumed that the two subpopulations are identical except for several parameters (Table 1). The model for each subpopulation is based on a previous model of inspiratory preBötC neurons (Toporikova and Butera, 2011), where the fast-spiking component has been removed (Rubin and Terman, 2002) and voltage-gated Ca^2+^ current has been added. Each compartment of the model represents a subpopulation of such preBötC neurons that are interconnected with predominantly excitatory synaptic coupling.

**Table 1:** Parameters for separate subpopulation models. The middle column contains parameters for the eupnea subpopulation. The parameters for the sigh subpopulation are identical to the eupnea subpopulation except for those listed in the right column

Parameter related to	Eupnea subpopulation	Sigh subpopulation (same as eupnea except following)
*I*_Na_*_P_*	*g*_Na_*_P_* = 2.5 nS; *V_h_* = −48 mV; *V_m_* = −40 mV; *s_h_* = 5 mV; *s_m_* = −6 mV; $\bar{\tau_{h}}$ = 10000 ms; *V*_Na_*_P_* = 50 mV	*g*_Na_*_P_* = 1.3 nS
*I*_Ca_*_N_*	*g*_Ca_*_N_* = 1.5 nS; K_Ca_*_N_* = 0.74 μM	
*I*_K_	*g*_K_ = 2.7 nS*; V*_K_ = −60 mV	
*I_h_*	*g_h_* = 2 nS; *sn* = 8 mV; *V_n_* = −90 mV; *V_H_* = 30 mV	*V_n_* = −70 mV
*I*_Ca_	*g*_Ca_ = 0.02 nS; *V*_Ca_ = 150 mV; α = 0.055; *V_PMCA_* = 2; K*_PMCA_* = 0.3	
ER Ca	λ = 0.0001; *f_i_* = 0.000025; [IP_3_] = 1 μM;*A* = 0.0005; $V_{SERCA}=400\frac{aMol}{s}$; K*_SERCA_* = 0.2; *σ* = 0.185; $L_{IP_{3}R}=\frac{0.37pL}{s}$; $P_{IP_{3}R}=31000\frac{pL}{s}$; K*_I_* = 1.0 μM; K*_d_* = 0.4 μM; K*_a_* = 0.4 μM	λ = 0.1
Synaptic	*g_syn_* = 9 nS; *V_syn_* = 0 mV; *V_ss_* = −10 mV; *S_S_* = −5 mV; $\bar{\tau_{s}}$= 5 ms; *k_syn_* = 1	*g_syn_* = 3 nS; *V_syn_* = −70 mV
Other	*Cm* = 21 pF	

The voltage of each compartment (*V*) is determined by using a balance of essential burst-generating currents (Toporikova and Butera, 2011): a voltage-gated persistent Na^+^ current (*I*_Na_*_P_*) and a K^+^-dominated passive leak current (*I*_leak_), a non-inactivating voltage-gated calcium current (*I*_Ca_), a voltage-gated hyperpolarization-activated current (*I_h_*), calcium-activated nonspecific cation current (*I_CaN_*), and a synaptic current (*I_syn_*). Therefore, the voltage component of the model is described by the following equation:

(1) $$C_{m}\frac{dV}{dt}=-I_{NaP}-I_{\mathrm{leak}}-I_{Ca}-I_{CaN}-I_{h}-I_{syn}$$

where the currents are given by:

(2) $$I_{NaP}=g_{NaP}m_{\infty }\;h(V-V_{NaP})$$

(3) $$I_{Ca}=g_{Ca}m_{\infty }\;(V-V_{Ca})$$

(4) $$I_{CaN}=g_{CaN}\frac{\left[Ca\right]}{\left([Ca]+K_{CaN}\right)}\;(V-V_{Nap})$$

(5) $$I_{\mathrm{leak}}=g_{K}(V-V_{K})$$

(6) $$I_{syn}=g_{syn}s(V-V_{syn})$$

(7) $$I_{h}=g_{h}n_{\infty }(V-V_{h})$$

Here, the parameter *g* represents the maximal conductance of each current (Na*P*, K, Ca*N*, Ca, *H*, or synaptic), while *m* and *n* are activation functions and *h* is the inactivation variable. To reduce the number of parameters in the model, the activation of *I*_Ca_ was approximated with the activation function for *I*_Na_*_P_*. This fast, non-inactivated current is intended to track average changes in Ca^2+^ during the burst. Specific parameters for *I_h_* were fitted to experimental activation curve in Figure 7*A*, except for reversal potential, which was chosen to fit experimental data on sigh shape in Figure 7*B*.

The synaptic activation variable *s* can be defined as:

(8) $$\frac{ds}{dt}=\frac{(1-s)H(V)-k_{syn}s}{\tau_{syn}}$$

where *k_syn_* is synaptic dissociation constant.

Inactivation variable *h* is described by the following equation:

(9) $$\frac{dh}{dt}=\frac{h_{\infty }(V)-h}{\tau_{h}}$$

where *h_∞_*(*V*) is the steady state activation/inactivation curve and *τ_h_* is the voltage-dependent time constant. The steady state activation/inactivation curves and synaptic activation function *H*(*V*) are modeled as a sigmoid, with *x* representing *m*, *n*, *s*, and *h*:

(10) $$x_{\infty }=\frac{1}{1+exp\left(\frac{V-V_{x}}{s_{x}}\right)}$$

while the time constant is modeled as follows

(11) $$\tau_{x}=\frac{\overset{¯}{\tau_{x}}}{cosh\left(\frac{V-V_{x}}{2s_{x}}\right)}$$

We used a two-pool model to account for Ca^2+^ fluxes through the cells’ plasma membrane and endoplasmic reticulum (ER). Therefore, Ca^2+^ kinetics are modeled by three equations representing the intracellular Ca^2+^ ([Ca]*_i_*) balance, ER Ca^2+^ ([Ca]*_ER_*) balance and IP_3_ channel gating variable (*l*) (Li and Rinzel, 1994).

(12) $$\frac{d{\left[Ca\right]}_{i}}{dt}=f_{i}\left(\frac{1}{\lambda }\left(J_{PM_{IN}}-J_{PM_{OUT}}\right)-\left(J_{ER_{IN}}-J_{ER_{OUT}}\right)\right)$$

(13) $$\frac{d{\left[Ca\right]}_{ER}}{dt}=\frac{f_{i}}{\sigma }(J_{ER_{IN}}-J_{ER_{OUT}})$$

(14) $$\frac{dl}{dt}=A(K_{d}-l({\left[Ca\right]}_{i}+K_{d}))$$

where λ is the ratio of ER to plasma membrane surfaces, $\lambda =\frac{A_{ER}}{A_{pm}}$, *f_i_* is a constant reflecting the fraction of bound-to-free Ca^2+^ concentration normalized to effective area (Wagner and Keizer, 1994), *A* is a scaling constant, *K_d_* is the dissociation constant for IP_3_ receptor inactivation by Ca^2+^, and σ is the ratio of cytosolic to ER volumes.

The flux into the cytosol from the ER ($J_{ER_{IN}}$) is regulated by the activity of IP_3_ receptors and is defined as:

(15) $$J_{ER_{IN}}=\left(L_{IP_{3}R}+P_{IP_{3}R}{\left[\frac{[IP_{3}]\;{\left[Ca\right]}_{i}\;l}{\left([IP_{3}]+K_{I})({\left[Ca\right]}_{i}+K_{a}\right)}\right]}^{3}\right)\;\times \;\left({\left[Ca\right]}_{ER}-{\left[Ca\right]}_{i}\right)$$

where $P_{IP_{3}R}$ is the maximum total permeability of IP_3_ channels, $L_{IP_{3}R}$ is the ER leak permeability, [*IP*_3_] is the *IP_3_* concentration, *K_I_* and *K_a_* are the dissociation constants for IP_3_ receptor activation by IP_3_ and Ca^2+^, respectively.

The flux from the cytosol back to the ER ($J_{ER_{OUT}}$) is controlled by the activity of sarco/endoplasmic reticulum Ca^2+^ ATPase (SERCA) pumps:

(16) $$J_{ER_{OUT}}=V_{SERCA}\frac{{\left[Ca\right]}_{i}^{2}}{K_{SERCA}{}^{2}+{\left[Ca\right]}_{i}^{2}}$$

where *V_SERCA_* is the maximal SERCA pump rate, *K_SERCA_* is the coefficient for the SERCA pumps.

The influx of Ca^2+^ through the plasma membrane is proportional to the voltage-gated Ca current

(17) $$j_{PM_{IN}}=-\alpha \;I_{ca}$$

where α is a proportionality constant.

The outflux of Ca^2+^ through the plasma membrane is controlled by the activity of PMCA pumps as follows:

(18) $$j_{PM_{OUT}}=V_{PMCA}\frac{c^{2}}{K_{PMCA}^{2}+c^{2}}$$

where *V_PMCA_* is the maximum activity of PMCA pumps and *K_PMCA_* is the kinetic constant for PMCA pumps.

The values for sigh and eupneic cell populations’ parameters are given in Table 1.

The differential equations were solved numerically using Python programming language. Numerical integration was carried out using *odeint* function of Scypy library with an adaptable time step. This method solves system of ordinary differential equations using *lsoda* method for stiff or nonstiff systems. To ensure that the model was in a stable oscillatory regime, the first 6 s of simulations were systematically discarded. (The source code for the model is available at http://modeldb.yale.edu/181962).

### Experimental methods

All animal procedures were performed in accordance with the authors’ university’s and the national Committee of Animal Care regulations. Experiments were performed on mouse embryos (either sex) that were obtained from pregnant OF1 females raised in our laboratory's breeding facility. To remain consistent, we performed our physiological experiments at the same developmental stage at which data used to build our model were obtained; i.e., embryonic day (E) 18.5.

#### Rhythmic slice preparation

**Figure 1:**
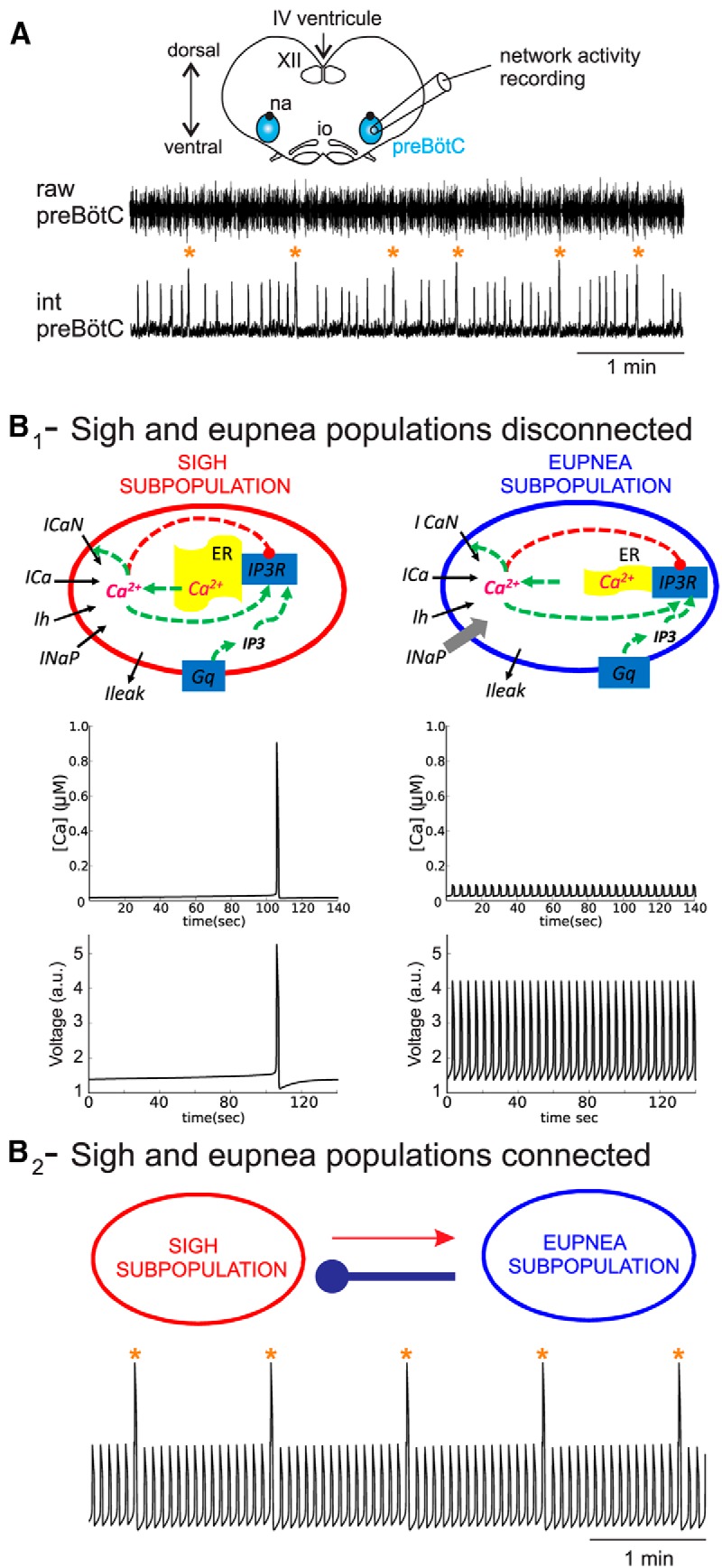
Sigh and eupnea activity patterns *in vitro* and *in silico*. ***A***, Schematic representation of an *in vitro* transverse brainstem slice preparation isolating the pre-Bötzinger complex respiratory network (preBötC). Raw (upper trace) and integrated (bottom trace) preBötC activity recordings were obtained with an extracellular macro-electrode positioned at the surface of the slice. ***B_1_***, Diagrams of sigh (left) and eupnea (right) network models. Except for parameter values for the ER capacity (λ), *I*_Na_*_P_*, and *I_h_*, the sigh and eupnea models are identical. Intracellular Ca^2+^ (top) and voltage (bottom) obtained for individual uncoupled compartments (*g_syn_* = 0). Left and right panels show outputs for sigh and eupnea compartments, respectively. ***B_2_***, Model with two compartments connected through an inhibitory synapse (blue) from eupnea to sigh and an excitatory synapse (red) from sigh to eupnea subpopulations. The difference in symbol thickness represents the difference in synaptic strength. Bottom trace shows the average voltage trace of the coupled model that is concomitantly generating sigh and eupnea bursts. Orange stars indicate sigh events. XII, Hypoglossal nucleus; io, inferior olive; na, nucleus ambiguus.

Embryonic brainstem transverse slices isolating the preBötC network were obtained using the following procedure: pregnant mice were killed by cervical dislocation on E18.5, the day of the plug being considered to be E0.5. Embryos were excised from their uterine bags, manually stimulated to trigger spontaneous breathing behavior and then kept under a slight heating light until their experimental use. Slice preparations were dissected in cold oxygenated artificial CSF (aCSF) composed of (in mM): 120 NaCl, 8 KCl, 1.26 CaCl_2_, 1.5 MgCl_2_, 21 NaHCO_3_, 0.58 NaH_2_PO_4_, 30 glucose, pH 7.4. First, the hindbrain was isolated from the embryo's body by a rostral section made at the level of the rhombencephalon and a caudal section made at the level of the first cervical roots (between C2 and C4). Second, the isolated hindbrain was placed in a low melting point agar block and carefully oriented in order to proceed to serial transverse sectioning from rostral to caudal, using a vibratome (Leica). A 450-µm-thick slice, with its anterior limit set 250-300 µm caudal to the more caudal extension of the facial nucleus, was isolated. Other anatomical landmarks such as the wide opening of the fourth ventricle, the presence of the inferior olive, the nucleus ambiguous, and the hypoglossal nucleus were also used to select the appropriate sectioning axis (as also referred to in newborn mice by Ruangkittisakul et al., 2011; see also Fig. 1*A*). Such slice preparations encompass the region containing a significant portion of the preBötC network capable of spontaneously generating rhythmic fictive inspiratory activities. Slices were then transferred to the recording chamber, rostral surface up, and continuously alimented with oxygenated aCSF at a temperature of 30 °C. Preparations were allowed to recover from the slicing procedure over a period of 20 min before any recording sessions were started.

#### Recording procedures

Recordings of local population activity in slices were performed using glass micropipettes (with a tip diameter of 80-100 µm) positioned at the surface of the slice on top of the preBötC network that was located ventral to the nucleus ambiguus (a structure visible when the slice is exposed to direct light from below; Fig. 1*A*). The micropipettes used as suction electrodes were fabricated from borosilicate glass tubes broken at the tip (Harvard Apparatus), filled with aCSF and connected to a high-gain amplifier (AM Systems). The signals were filtered (bandwidth between 3 Hz and 3 kHz), integrated (time constant 100 ms; Neurolog, Digitimer), and recorded on a computer through a Digidata 1440 interface and with the PClamp10 software (Molecular Devices). The stored files were analyzed offline.

Whole cell patch-clamp recordings were performed under visual control using differential interference contrast. Patch pipettes were fabricated with borosilicate glass tubes using a puller (Sutter Instrument) and filled with a solution composed of the following (in mM): 140 K-gluconate acid, 1 CaCl_2_·6H_2_O, 10 EGTA, 2 MgCl_2_, 4 Na_2_ATP, 10 HEPES (pH 7.2); and had tip resistances of 5-7 MΩ when filled with this solution. Electrophysiological signals were recorded using an Axoclamp 2A amplifier (Molecular Devices) and the same digitizing interface and software as described above. Recorded neurons were all located close to the extracellular pipette and were selected on the basis that their discharge was in phase with the population activity (see Fig. 7*A*). The *I_h_* current was activated by applying 2 s duration 10 mV incrementing hyperpolarizing voltage steps from a holding potential of −50 mV. To trace the corresponding *I*/*V* curve, the amplitude of *I_h_* was obtained by subtracting the current amplitude measured at the end of the voltage step from the current measured at the beginning of the voltage step.

#### Calcium imaging

The activity of multiple preBötC neurons was simultaneously monitored using calcium imaging. Slice preparations were first incubated in the dark for 45 min at room temperature in a solution of oxygenated aCSF containing the cell-permeable calcium indicator dye Calcium Green-1 AM (10 µM; Life Technologies). After loading, preparations were positioned in the recording chamber, rostral side up. A subsequent 30 min delay was observed to wash out the excess dye and for the preparation to stabilize in oxygenated aCSF at 30 °C. Fluorescent signals were captured through a FN1 upright microscope (Nikon) equipped with an epifluorescent illumination system and a fluorescein filter coupled to an Exiblue camera (QImaging). Images (100 ms exposure time) were acquired over periods lasting 120 s and analyzed using software developed by Dr N. Mellen (Mellen and Tuong, 2009).

#### Pharmacology

Drugs, obtained from Sigma or Tocris, were dissolved in aCSF or DMSO and bath-applied for 15-30 min at their final concentration of 1 µM for strychnine; 4 µM for cadmium; 10-20 µM for Riluzole; 50 µM for ZD7288, an *I_h_* blocker; 1-10 µM for thapsigargin; 50-100 µM for cyclopiazonic acid (CPA); and 5-10 µM for ryanodine. The drug effects were measured at the end of the exposure period and frequency values are given as mean ± SEM. Statistical significance was assessed by Student’s *t* test, Mann−Whitney, or one-way ANOVA, as appropriate. Mean values were considered to be significantly different at *p* < 0.05.

## Results

### A two-compartment computational model of the preBötC network generates eupneic- and sigh-like activities

In embryonic brainstem slice preparations (Fig. 1*A*, top), the preBötC respiratory network generates two distinct patterns of rhythmic activity (Fig 1*A*; see also Chapuis et al., 2014), as also observed in newborn slices (Lieske et al., 2000). Extracellular population recordings showed the expression of sigh-like bursts at a low frequency (1.3 ± 0.1 burst/min, *n* = 15) and with a large amplitude (Fig. 1*A*, traces, orange stars) and eupnea-like bursts with higher frequency (16.3 ± 0.5 burst/min, *n* = 15) and smaller amplitude (Fig. 1*A*, traces, bursts without stars). Each sigh episode was followed by an extended period of eupnea (mean duration 8.4 ± 0.3 s, *n* = 7), a delay referred to as post-sigh apnea. Both fictive activities were generated spontaneously and concomitantly in control conditions.

We developed a two-compartment mathematical model of the preBötC network that reproduced the pattern of *in vitro* fictive sigh and eupneic activities. Each compartment of the model represents a group of preBötC neurons connected predominantly through excitatory synapses. Then the rhythmic activity in each compartment reflects a combination of cellular properties and intercellular connections. The sigh and eupnea compartments of the model are identical except for conductance of a persistent sodium current (*g*_Na_*_P_*), ER capacity expressed as a ratio of ER membrane to plasma membrane areas (λ), and activation threshold of a hyperpolarization-activated current (*I_h_*), (Fig. 1*B1*, diagrams). All other parameters of the model can be found in Table 1. Based on inspiratory-related activity features observed in *in vitro* recordings, we assumed that each compartment possesses distinctly different rhythm-generating mechanisms. We hypothesized that the slow sigh rhythm results mainly from slow Ca^2+^ oscillations, whereas the fast eupnea rhythm follows mainly the kinetics of the persistent Na^+^ current (*I*_Na_*_P_*), since it has been shown that the preBötC activity relies heavily upon *I*_Na_*_P_* (Del Negro et al., 2002; Peña et al., 2004; Del Negro et al., 2005; Koizumi and Smith, 2008). To demonstrate the independent oscillations in each compartment, we first plotted the voltage and intracellular Ca^2+^([Ca]*_i_*) of each compartment in an uncoupled model ($g_{sym_{INH}}=g_{syn_{STIM}}=0$) as depicted in Figure 1*B1*. The voltage oscillations in the sigh compartment followed changes in intracellular Ca^2+^ periodically released out of ER (Fig. 1*B1*, left two panels). The slow activation of *I_h_* led to a depolarization and activation of voltage-gated Ca^2+^ current, *I*_Ca_, which eventually activated inositol 1,4,5-triphosphate receptors (IP_3_R) on the ER membrane, thereby triggering Ca^2+^ release into the cytosol. The slow inactivation of IP_3_R by Ca^2+^ terminated the Ca^2+^ oscillation and induced a prolonged period of hyperpolarization (post-sigh apnea) during which SERCA pumps transported Ca^2+^ back into the ER. The voltage oscillations in the eupnea compartment followed the kinetics of *I*_Na_*_P_* (Fig. 1*B1*, diagram). This current exhibits a fast activation and slow inactivation, which can induce bursting activity in a preBötC model neuron (Butera et al., 1999; Del Negro et al., 2005; Koizumi and Smith, 2008). In contrast to the oscillations in the sigh compartment, Ca^2+^ influx in eupnea compartment did not play a rhythmogenic role. Although depolarization-activated *I*_Ca_ led to an influx of Ca^2+^ during each eupnea oscillation, the ER capacity in this model was not large enough to induce Ca^2+^ release from the ER. Thus, each compartment produced its own intrinsic rhythmic activity: the eupnea compartment generated high-frequency, low-amplitude oscillations while the sigh compartment generated low-frequency, high-amplitude oscillations (Fig. 1*B1*, bottom panels). We chose relatively short cycle periods for sigh rhythmicity (1.2 min) to better illustrate the shape of the global output. However, the cycle period of sighs could be extended up to 5 min by varying the combination of *g*_Na_*_P_* and *g*_Ca_ (data not shown).

Next, we established synaptic connections between the two compartments and found that with an inhibitory synapse from the eupneic subpopulation to the sigh subpopulation and an excitatory synapse from the sigh to eupneic subpopulations (Fig. 1*B2*, diagram), the output of the model became very similar to the one recorded *in vitro* (compare bottom traces in Fig. 1*A*, and *B2*). We also found that the weight of the inhibitory synaptic influence from the eupneic to sigh subpopulations has to be set considerably higher than the reciprocal excitatory one (see Discussion, below). With these parameters, large-amplitude low-frequency bursts (Fig. 1*B2*, orange stars) were intermingled with small-amplitude, high-frequency eupneic-like bursts (Fig. 1*B2*). The frequencies of the sigh and eupnea rhythms were 0.85 and 13.9 bursts/min, respectively, which presents a temporal relationship that is similar to that seen in the experimental data (Fig. 1*A*). Although shorter than in *in vitro* recordings, the model expressed a theoretical post-sigh apnea, consisting of a 23% increase in the duration of the eupneic period immediately after the sigh (5.34 s post-sigh burst compared to 4.32 s post-eupnea burst). In contrast, when we tried to connect the two compartments through other combinations of synaptic interactions (i.e., reciprocally inhibitory, reciprocally excitatory, and inhibitory from sigh to eupnea subpopulations) the model failed to reproduce the typical eupnea-sigh discharge pattern found experimentally (data not shown and see below). Therefore, with two compartments possessing distinct endogenous cellular properties and connected through a specific combination of inhibitory and excitatory synapses, our model can mimic the bimodal motor burst rhythms of the biological preBötC network.

### The model reproduces the changes in sigh burst shape after the blockade of inhibitory synapses

**Figure 2:**
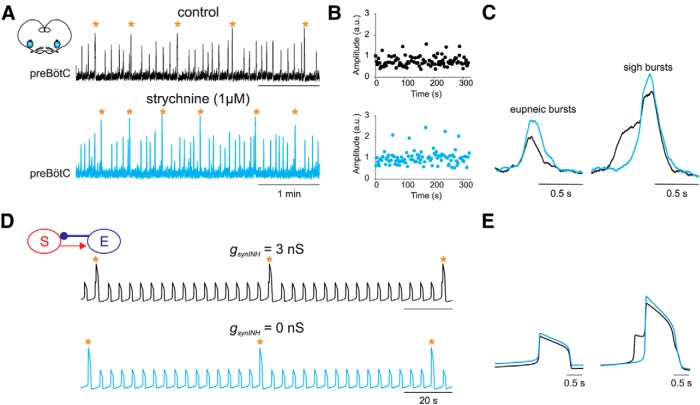
Biphasic shape of sigh bursts requires inhibitory synaptic input. ***A−C***, *In vitro* recordings of sigh and eupnea activities in control conditions and after blockade of glycinergic synapses. ***A***, Integrated traces of preBötC recordings in control conditions (black, top) and in the presence of 1 µM strychnine (blue, bottom). Both recordings show two types of bursts corresponding to eupnea and sigh. ***B***, Sequential slots of the amplitude (in arbitrary units) of inspiratory bursts versus time in control conditions (top) and under strychnine (bottom). Note that the amplitudes of both types of burst were significantly larger in the presence of strychnine. ***C***, Averaged traces for eupneic bursts (*n* = 10; left) and sigh bursts (*n* = 8; right) in control (black) and strychnine (blue) conditions. ***D***, *In silico* average voltage output of a two-compartment coupled model with (top trace) and without (bottom trace) synaptic inhibition ($g_{syn_{INH}}$). ***E***, Average profile of eupnea (left) and sigh bursts (right) obtained from traces in ***D***. Orange stars indicate sigh events.

In addition to frequency and amplitude differences, sigh motor output events differ from eupneic bursts in terms of shape (Figs. 1, 2*A*), (Lieske et al., 2000; Tryba et al., 2008; Chapuis et al., 2014). Whereas eupneic activity has a monophasic shape, sighs are defined as biphasic bursts with the initial phase being comparable to a eupneic event and the second phase having larger amplitude (Fig. 2*C*, black trace). However, as previously published (Lieske et al., 2000; Chapuis et al., 2014) and illustrated in Figure 2*A*, the two components of a sigh burst can be separated by blockade of inhibitory glycinergic synaptic connections within the preBötC network. We confirmed this finding in embryonic mouse brain stem preparation *in vitro* by bath application of strychnine (1 µM), which rendered the high-amplitude, low-frequency events monophasic in shape (Fig. 2*C*, blue trace). Blockade of glycinergic synapses also induced an increase in amplitude of both eupneic and sigh bursts (Fig. 2*B*,*C*), suggesting that inhibitory glycinergic inputs are able to limit the overall magnitude of bursting activity throughout the inspiratory neuronal population.

We then used the outcome of the strychnine experiments to narrow options for the connections between the sigh and eupnea compartments in our model. By testing *in silico* four possible connections between the two compartments (all results not shown), we found that only one of these configurations reproduced the observations made in the *in vitro* strychnine experiments (Fig. 2*D*): when eupnea inhibited sigh and sigh excited eupnea, the sigh burst shape was biphasic, similar to the experimental *in vitro* recordings (Fig. 2*D*, top trace). When the inhibitory connection between the sigh and eupnea compartments was blocked ($g_{syn_{INH}}=0$), the sigh shape became monophasic (Fig. 2*D*, bottom trace; 2*E*, right), while the average shape of eupnea oscillations remained unchanged (Fig. 2*E*, left). It should be noted that the data from the strychnine experiment could only be reproduced for a specific ratio of inhibition to excitation of 1:3 ($g_{syn_{STIM}}=9\text{nS}$, $g_{syn_{INH}}=3\text{nS}$). Therefore, the biphasic sigh shape in our model reflected the strength of coupling between the two compartments and required both a weaker inhibitory synapse from the eupnea subpopulation to the sigh subpopulation and a stronger reciprocal excitatory connection.

### Sighs but not eupnea are abolished by a reduction of calcium conductances and disruption of ER activity

**Figure 3:**
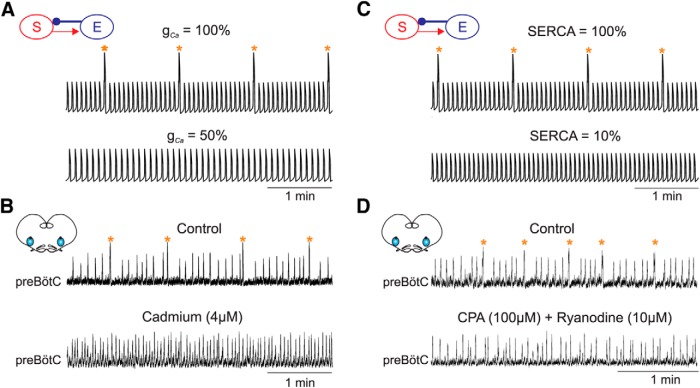
Calcium-dependent mechanisms are critically involved in sigh but not eupnea generation. ***A***, *In silico*: Average model voltage with control value (*g*_Ca_ = 0.02 nS, top trace) and with 50% reduction (*g*_Ca_ = 0.01 nS, bottom trace) of calcium conductance. ***B***, *In vitro*: Extracellular recordings of preBötC activity in control conditions (top trace) and after bath application of 4 µM cadmium to impair calcium conductances (bottom trace). A partial blockade of calcium conductances *in silico* and *in vitro* specifically prevents sigh bursts generation. ***C***, *In silico*: Average model voltage with control value (top trace) and with 90% reduction (bottom trace) for SERCA activity. ***D***, *In vitro*: Extracellular recordings of preBötC activity in control conditions (top trace) and after bath application of 100 µM CPA + 10 µM ryanodine to impair ER activity (bottom trace). A significant blockade of ER activity *in silico* and *in vitro* specifically prevents sigh bursts generation. Orange stars indicate sigh events.

In our model, activity in the eupnea compartment depends mainly upon the activation of *I*_Na_*_P_* while sigh compartment activity relies on a specific intracellular Ca^2+^ mechanism that combines the activation of membrane conductances (*I*_CaN_ and *I*_Ca_) and second messenger pathways including the ER machinery. Because the intracellular source of calcium does not contribute significantly to eupneic rhythm generation in the preBötC (Beltran-Parrazal et al., 2012) and given the design of our sigh compartment, we expected that partially blocking voltage-gated Ca^2+^ input would affect rhythmogenesis of sigh but not eupnea. We simulated this scenario *in silico* by reducing the voltage-gated Ca^2+^ conductance (*g*_Ca_) to 50% of its original value. The resulting population activity is shown in Figure 3. Compared to control (Fig. 3*A*, top), a reduction in *g*_Ca_ removed sighs completely, but did not significantly affect eupnea oscillations (Fig. 3*A*, bottom). To test the physiological correlate, we conducted equivalent *in vitro* experiments by applying 4 µM cadmium (Cd), which at this low concentration allows partial blockade of a large spectra of unspecified calcium currents (Elsen and Ramirez, 1998). Cd application selectively prevented fictive sigh burst occurrence while inducing a slight but significant increase in the frequency of ongoing fictive eupneic bursts (15.5 ± 0.2 burst/min in control vs 18.4 ± 0.2 burst/min in Cd; *n* = 8, *p* < 0.005; Fig. 3*B*). These results are consistent with our model and with previous findings showing that sigh generation in the newborn mouse is critically dependent upon the activation voltage-gated calcium channels and more specifically the P/Q-type (Lieske et al., 2000; Lieske and Ramirez, 2006a; Koch et al., 2013).

In a second series of experiments, we investigated whether disrupting Ca^2+^ sequestration and release from the ER would more specifically affect sigh than eupnea activity. We tested this both *in silico* and *in vitro* and results obtained are presented in Figure 3. In order to significantly affect ER activity *in vitro*, we simultaneously targeted the calcium fluxes coming from and going to the ER by co-applying thapsigargin (10 µM; *n* = 2) or CPA (100 µM; *n* = 3) and ryanodine (10 µM). We observed that reducing SERCA activity down to 10% in the model (Fig. 3*C*) or blocking ER activity using pharmacological agents *in vitro* (Fig. 3*D*; *n* = 5) prevented sigh from being generated. In such conditions, eupnea was the only inspiration-related activity detectable and was generated at a higher frequency compared to control (11.3 ± 0.8 burst/min in control vs 17.5 ± 0.6 burst/min in SERCA blocked; *n* = 5, *p* < 0.001). Note that when the drugs were applied at lower concentrations (<10 µM for thapsigargin and ryanodine and <100 µM for CPA; *n* = 6) or when SERCA activity was kept higher than 10% of its maximal value in the model, sighs were still generated (data not shown), suggesting that a minimal ER activity can sustain sigh generation. Altogether, the outcome of our combined *in vitro* and *in silico* experiments supports our assumption that Ca^2+^ entry through voltage-gated channels, together with the intracellular calcium handling machinery, are major components of the sigh-generation mechanisms.

### Sigh and eupnea are sensitive to *I*_Na_*_P_* blockade

**Figure 4:**
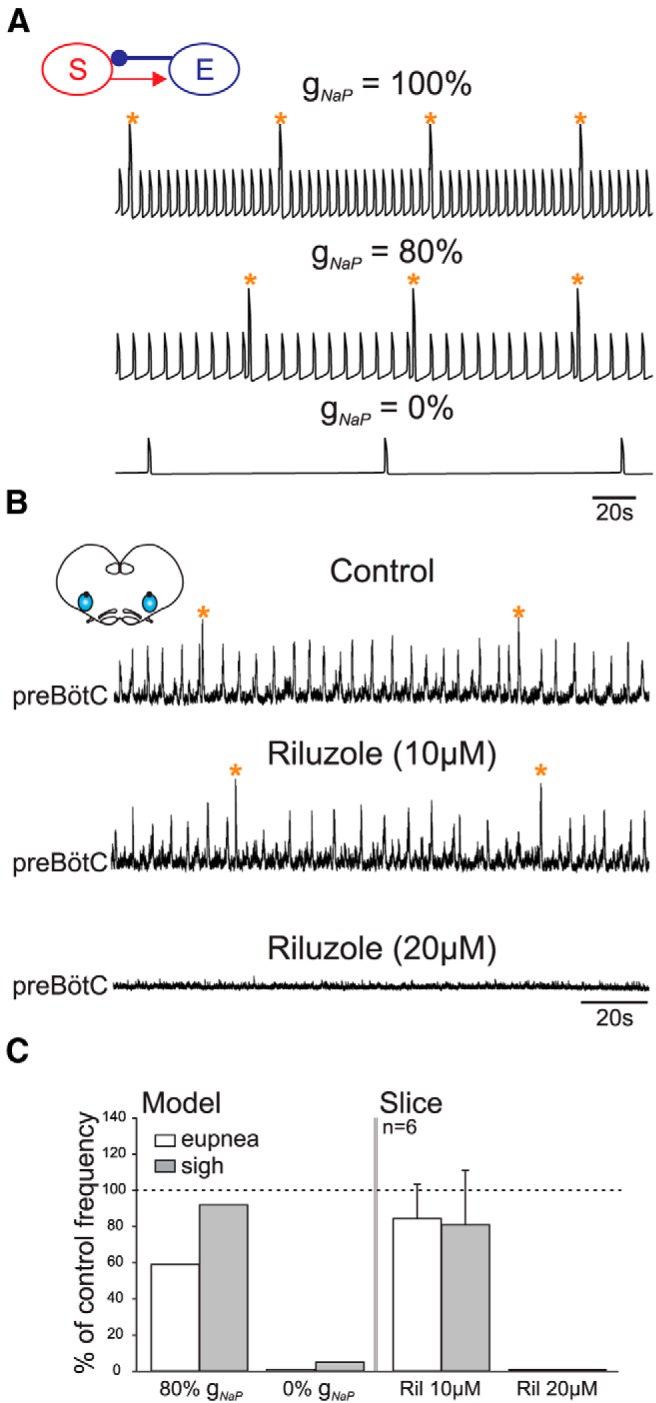
A persistent sodium current is critically involved in sigh and eupnea generation. ***A***, *In silico* experiment showing the effects of reducing *g*_Na_*_P_* from 100% (2.5 nS for eupnea and 1 nS for sigh, top trace) to 80% (2 nS for eupnea and 0.8 nS for sigh, middle trace) and to 0% (bottom trace) on global network activity. ***B***, *In vitro*: Extracellular recordings of preBötC activity in control conditions (top trace) and under increasing concentrations of riluzole (middle and bottom traces) to impair the persistent sodium current. ***C***, Quantification of burst frequency changes under the partial and full blockade of *g*_Na_*_P_* for fictive eupnea (white bars) and sighs (gray bars) in the model (left) and *in vitro* (right). Orange stars indicate sigh events.

We acknowledge that both I_Ca_*_N_* and I_Na_*_P_* are important in respiratory rhythmogenesis (Del Negro et al., 2002; Peña et al., 2004; Del Negro et al., 2005; Pace et al., 2007a,b); however, in the present version of our model, we mainly examined the potential role of *I*_Na_*_P_* in sigh generation only. Although *I*_Na_*_P_* is expressed in the sigh compartment of our model, its value was set lower than the one required for stable oscillations. In contrast, the generation of rhythmic activity in the eupnea compartment relies mainly on the persistent sodium conductance (*g*_Na_*_P_*). Consequently, a reduction of *g*_Na_*_P_* should affect the eupnea rhythm more than the sigh rhythm. We therefore tested the involvement of *I*_Na_*_P_* in rhythmic activity *in silico* by progressively reducing *g*_Na_*_P_*. As expected, a decrease in *g*_Na_*_P_* (*g*_Na_*_P_* = 80% of control) reduced the frequency of eupnea activity without much effect on the sigh frequency (Fig. 4*A*,*C*). Complete removal of *I*_Na_*_P_* abolished oscillations in the eupnea compartment. Since in the sigh compartment, *g*_Na_*_P_* is activated only during Ca^2+^ oscillations, its removal decreased the size of sigh bursts that became indistinguishable in amplitude from typical eupneic bursts.

We conducted comparable *in vitro* experiments by blocking *I*_Na_*_P_* using Riluzole (Ril; 10-20 *µ*M; *n* = 6). In five preparations, exposure to 10 µM Ril had little effect on either sigh or eupnea burst frequencies (*p* = 0.6). However, when applied at 20 µM, Ril completely blocked both fictive rhythms (Fig. 4*B*,*C*). In the remaining preparation, both eupnea and sigh activities were blocked in the presence of 10 µM Ril. These findings therefore suggest that, in the embryo, *in vitro* sigh and eupneic rhythm generation rely to a qualitatively similar extent on *I*_Na_*_P_* expressed within inspiratory preBötC neurons. These results also show that, in the embryo, *I*_Na_*_P_* plays a major role in respiratory rhythmogenesis, while the *I*_Ca_*_N_* current, being unable to sustain the rhythm when *I*_Na_*_P_* is blocked, very probably does not play a critical role in rhythm generation, in contrast to what is considered at postnatal stages (Del Negro et al., 2002; Peña et al., 2004; Del Negro et al., 2005; Pace et al., 2007a,b). Thus, our combined *in vitro* and *in silico* experiments suggest that, in the embryo, both sigh and eupnea rhythm generation relies on *I*_Na_*_P_*, with the distinction that *g*_Na_*_P_* is directly involved in the rhythmogenic mechanisms of the eupnea subpopulation, whereas in the sigh subpopulation, it serves to amplify already existing depolarizing potentials in each cycle.

### Sigh frequency is insensitive to changes in extracellular K^+^ concentration

**Figure 5:**
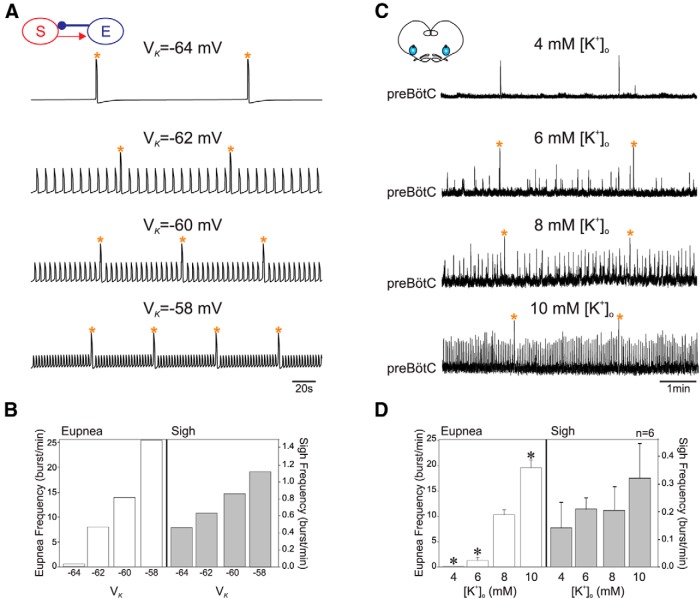
Eupneic activity is more sensitive to [K^+^]*_o_* than sigh activity. ***A***, Voltage traces of *in silico* experiments where *V*_K_, which is directly correlated to [K^+^]*_o_* was progressively increased (from top to bottom). ***B***, Bar plots representing the mean frequency for eupnea (left, unfilled bars) and sigh bursts (right, shaded bars) for different *V*_K_. ***C***, Extracellular recordings of spontaneous preBötC activity from slice preparation bathed in aCSF containing 4, 6, 8, and 10 mM of [*K* ^+^]*_o_* (respectively, from top to bottom). ***D***, Histograms of mean (+SEM) eupneic (unfilled bars) and sigh (shaded bars) burst frequencies for different [K^+^]*_o_*. Sigh bursts were generated at a relatively constant frequency whereas the eupnea burst frequency increased with increasing [K^+^]*_o_* both *in silico* and *in vitro*. *p < 0.05. Orange stars indicate sigh events.

We hypothesized that sighs are generated mainly through ultra-slow (order of minutes) [Ca^2+^]*_i_* oscillations. In our model, such metabolic oscillations rely on periodic Ca^2+^ release from ER store. Although depolarization-activated Ca^2+^ influx through voltage-gated Ca^2+^channels might participate to the generation of these oscillations, the release from ER is only weekly voltage-dependent and thus depolarization should not significantly affect the frequency of such metabolic oscillations. In contrast, oscillations in the eupnea compartment reflect depolarization-dependent kinetics of *I*_Na_*_P_* and its frequency should directly reflect the changes in depolarization. Therefore, we expected that oscillations in the eupnea compartment of our model would be more sensitive to external K^+^ concentration ([K^+^]*_o_*) than oscillations in the sigh compartment. We tested the effect of changing [K^+^]*_o_* on the sigh and eupnea rhythm frequencies *in silico* by progressively increasing the reversal potential for the K^+^ dominated leak current (*V*_K_). Figure 5*A* shows average voltage profiles of the eupnea−sigh model for four different values of *V*_K_. For *V*_K_ = −64 mV, the eupnea compartment was below oscillatory threshold but the sigh compartment was still able to generate slow Ca^2+^ oscillations (0.4 burst/min). Thus, the model produced only the ultra-slow sigh oscillations and no eupneic activity (Fig. 5*A*, top panel). Note that this feature might be specific of the developmental stage examined here, since this is not observed at postnatal ages (Ruangkittisakul et al., 2011). As *V*_K_ increased (*V*_K_ = −62 mV), the eupnea compartment reached its threshold and the two-compartment model produced both sigh and eupnea oscillations (Fig. 5*A*, second panel). The eupnea frequency was 8.1 burst/min and the sigh frequency increased slightly to 0.6 burst/min. A further increase in *V*_K_ to its control value (*V*_K_ = −60 mV) doubled the eupnea frequency (14.8 burst/min), but the frequency of sighs increased only slightly to 0.8 burst/min (Fig. 5*A*, third panel). For a relatively depolarized value of *V*_K_ = −58 mV, we observed a much greater increase in the eupnea frequency compared to sighs, with the former increasing geometrically to 25 burst/min, and the latter increasing linearly to 1.1 burst/min (Fig. 5*A*, bottom panel). These data clearly demonstrate that for different levels of *V*_K_, the range of frequency increase in the eupnea compartment is significantly greater than that of the sigh compartment (Fig. 5*B*).

To test our modeling prediction *in vitro*, we applied different concentrations of [K^+^]*_o_* to brainstem slices in order to change overall levels of neuronal excitability while recording the integrated network output (Fig. 5*C*). We monitored six slice preparations, which spontaneously produced both fictive eupnea and sigh rhythms under the standard 8 mM [K^+^]*_o_* (Fig. 5*C*). In all six preparations, decreasing [K^+^]*_o_* to 4 mM caused eupneic bursting to cease completely while sigh bursts continued to be generated without any significant decrease in their cycle frequency (0.20 burst/min in control vs 0.14 burst/min in 4 mM [K^+^]*__o_*; *p* = 0.6; Fig. 5*C*,*D*). At 6 mM [K^+^]*_o_*, however, weak eupneic activity was also present (Fig. 5*C*) and thereafter showed a significant increase in burst frequency with increasing [K^+^]*_o_* (1.27 burst/min in 6 mM [K^+^]*__o_*, 10.32 burst/min in 8 mM [K^+^]*__o_*, and 19.6 burst/min in 10 mM [K^+^]*__o_*; *p* < 0.05; Fig. 5*C*,*D*). In contrast, sigh bursts were expressed under all [K^+^]*_o_* tested (Fig. 5*C*,*D*) and at frequencies that remained statistically unchanged (0.21 burst/min in 6 mM [K^+^]*__o_* and 0.32 burst/min in 10 mM [K^+^]*__o_*; *p* = 0.9 and 0.5, respectively; Fig. 5*D*). These data therefore indicate that excitability levels within the preBötC network have differential consequences for the generation of the two types of inspiratory activity, with the production of the slower sigh rhythm requiring less neuronal excitability than the expression of eupneic bursting. Accordingly, our *in vitro* experiments confirm our modeling prediction that frequency of eupnea is more sensitive to [*K* ^+^]*_o_* than sighs. Moreover, these results support the modeling assumption that sighs are generated through slow, voltage-insensitive Ca^2+^ oscillations, whereas eupnea relies mainly on voltage-sensitive ionic conductances.

### PreBötC activity in low [K^+^]*_o_* involves both neuronal subpopulations

**Figure 6:**
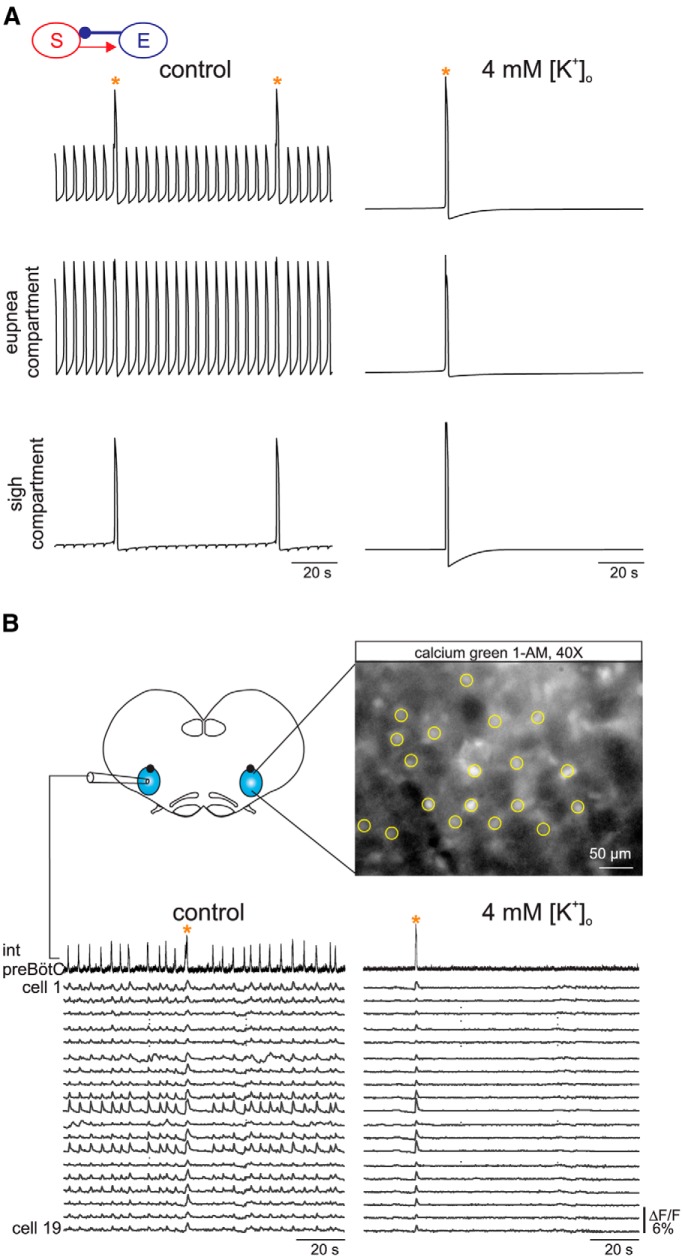
PreBötC activity in low [K^+^]*_o_* implicates neuronal activity from both subpopulations. ***A***, Voltage traces (top: average; middle: eupnea compartment; bottom: sigh compartment) of an *in silico* experiment in control conditions (left) and with *V*_K_ set low (right). ***B***, Schematic representation of the *in vitro* slice preparation from which an electrophysiological recording of preBötC activity was made simultaneously with calcium imaging performed on the contralateral respiratory network. Right, Image of a Ca^2+^-dye loaded slice obtained with a 40× objective. Yellow circles indicate rhythmically active cells. Bottom, Paired recordings of population electrical activity (top traces) and calcium transients (Δ*F*/*F*, bottom traces) in individual neurons (cells 1 to 19). All the cells displayed fluorescent changes synchronized to rhythmic electrical activity recorded on the contralateral side during both eupnea and sigh bursting in control conditions and during sigh-only activity in low [K^+^]*_o_*. Orange stars indicate sigh events.

Our simulations indicated that in low [K^+^]*_o_*, the network operated under a sigh-only regime. On this basis, we reasoned that the low [K^+^]*_o_* constitutes an experimental condition that allows the specific investigation of sigh-only neuron behavior. To test this, we examine the activity of the sigh and eupnea compartments of the model individually under low [K^+^]*_o_*; i.e., *V*_K_ = −64 mV. Unexpectedly, both subpopulations were active in phase in low extracellular [K^+^] (Fig. 6*A*). This effect could be explained by the presence of the model’s excitatory synaptic connection from the sigh to eupnea compartments. To test whether this was merely an artifact of our model, we ran *in vitro* experiments (*n* = 5) in which we examined the activity of several neurons simultaneously in control conditions ([K^+^]*_o_* = 8 mM) and in low [K^+^]*_o_* (4 mM) using calcium imaging. Consistent with our modeling results, we observed that all neurons active during fictive eupnea and sigh activities in control conditions were also active in low [K^+^]*_o_* (160 cells from five preparations, a mean of 32 cells per slice; Fig. 6*B*). Together, these experiments demonstrate that the synapse from the sigh subpopulation onto the eupnea subpopulation is strong enough to excite the entire network in low-excitability conditions. These results also indicate how intricately intermingled the two subpopulations are and hence the difficulty with which one can be functionally distinguished from the other.

### Sigh generation requires *I_h_* current activation

**Figure 7:**
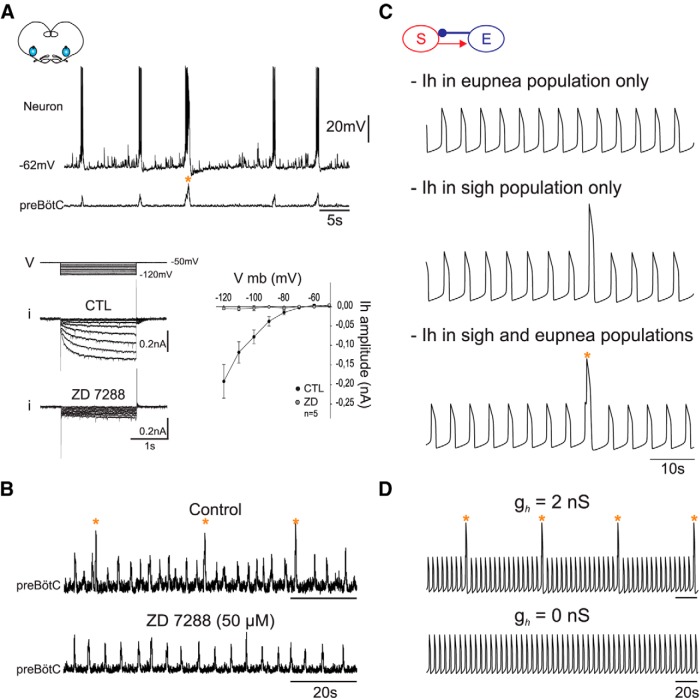
*I_h_* activation is required for sigh but not for eupnea generation. ***A***, Top, Simultaneous current-clamp recording of an inspiratory neuron (top trace) with integrated preBötC activity recording (bottom trace). Bottom left, Currents recorded in voltage clamp (bottom trace sets; i) evoked by hyperpolarizing voltage steps (top trace set; V) from a holding potential of −50 mV in control conditions (CTL) and in the presence of 50 µM ZD 7288 to block *I_h_*. Bottom right, Amplitude of *h* current versus membrane potential under control conditions (black circles) and in the presence of ZD 7288 (gray circles). *I_h_* was fully blocked by ZD 7288. ***B***, *In vitro* extracellular recordings of preBötC activity in control (top trace) and in the presence of 50 µM ZD 7288. ***C***, *In silico* experiments that included *I_h_* in the eupnea compartment model only (top), the sigh compartment only (middle), and in both compartments (bottom). The sigh bursts exhibit a biphasic shape only when *I_h_* is present in both compartments. ***D***, Voltage traces of *in silico* experiment with changes in *I_h_* from its control value *g_h_* = 2 nS, (top) to *g_h_* = 0 nS (bottom). Both *in silico* and *in vitro* blockade of *I_h_* selectively prevents sigh-burst generation. Orange stars indicate sigh events.

The hyperpolarization-activated inward *I_h_* is a conductance reported to play an important role in regulating respiratory cycle frequency (Thoby-Brisson et al., 2000), but its specific role in sigh generation has never been examined. To address this, we first tested whether *I_h_* was expressed in embryonic neurons generating both eupnea and sigh. We recorded 44 inspiratory neurons in voltage clamp conditions and found that the vast majority (*n* = 38; i.e., 86.4%) did indeed express the *I_h_* current. The conductance started to activate around −65 mV and was effectively blocked by ZD 7288 (50 µM) (Fig. 7*A*). Next, we determined *in vitro* whether *I_h_* was involved in fictive sigh generation by blocking its conductance in slices where the preBötC network exhibited a bimodal (eupnea and sigh) discharge pattern. In all preparations examined (*n* = 10), blocking *I_h_* with 50 µM ZD 7288 selectively prevented the generation of fictive sighs (Fig. 7*B*), while eupneic activity persisted, although at a lower frequency (13.8 ± 0.4 burst/min in control vs 12.3 ± 0.4 burst/min in ZD 7288; *n* = 10, *p* < 0.05). Comparable results were also found using the less specific *I_h_* blocker, caesium (5 mM; *n* = 4; data not shown). Together, these data indicate that, unlike eupneic activity, the induction of sigh bursts is critically dependent on *I_h_*.

Since *I_h_* blockade preferentially affects sighs *in vitro*, it is possible that this current is expressed only in the sigh subpopulation. To determine the distribution of *I_h_* between the two compartments of the model, we tested three scenarios *in silico*: *I_h_* expressed in (1) the eupnea subpopulation only, (2) the sigh subpopulation only, or (3) both. The simulation where *I_h_* was expressed solely in the eupnea compartment yielded no sigh activity (Fig. 7*C*, top trace). The simulation where *I_h_* was expressed in the sigh compartment only produced sighs but with a monophasic shape (Fig. 7*C*, middle trace). This is probably the result of weaker synchronization between eupnea and sigh, due to lower eupneic depolarization and reduced inhibition of sigh. Only when *I_h_* was expressed in both compartments were sigh bursts biphasic (Fig. 7*C*, bottom trace). In accordance with our patch clamp recordings, these results demonstrate that a high percentage of inspiratory neurons in the preBötC network express *I_h_* (see above) and that it is not a unique property of sigh cells. In addition, when we simulated *I_h_* blockade *in silico* by setting *g_h_* = 0, sigh bursting in our model was abolished and the model generated only eupnea activity (Fig. 7*D*). Therefore, our modeling data are consistent with the expression of *I_h_* in both eupnea and sigh subpopulations and confirms that *I_h_* activation is essential for sigh generation.

### Voltage-insensitivity of sigh activity is determined by *I_h_* activation threshold

**Figure 8:**
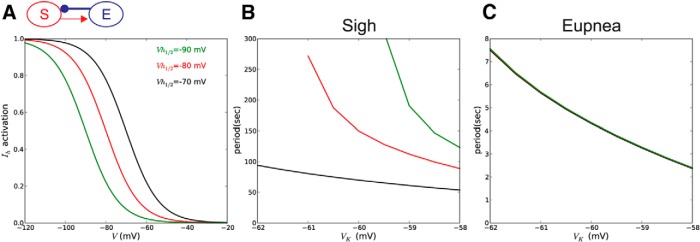
The [K^+^]*_o_* insensitivity of sighs is linked to *I_h_* activation characteristics. ***A***, Model tested with three activation curves for *I_h_* in the sigh compartment ($V_{h_{1/2}}=-90\text{mV}$, green;$V_{h_{1/2}}=-80\text{mV}$, red; $V_{h_{1/2}}=-70\text{mV}$, black). The *I_h_* activation curve for eupnea was kept constant at −90 mV. ***B***, The cycle period of sighs in response to different levels of *V*_K_ was measured for three levels of *I_h_* half-activation. The lowest half-activation is less sensitive to changes in *V*_K_. ***C***, The period of eupnea does not depend on *I_h_* half-activation.

The results of our *V*_K_ experiments clearly indicate that sigh oscillations are only weakly sensitive to [K^+^]*_o_*. However, the Ca^2+^ oscillations in the sigh compartment rely partially on voltage-gated Ca^2+^ influx for the initiation of Ca^2+^ release from the ER, and thereby should be somewhat sensitive to depolarization. Therefore, we suspected that some additional mechanism(s) is (are) responsible for the voltage-insensitivity of the sigh subpopulation. Since *I_h_* is involved in the slow depolarization of the sigh compartment, it is possible that this current was responsible for the compartment’s excitability insensitivity. To test this hypothesis, we ran *in silico* simulations to identify the consequences of changes in [K^+^]*_o_* in sigh generation in relation to *I_h_* activation threshold in the sigh compartment (Fig. 8*A*). When *I_h_* half-activation in sigh compartment was the same as in eupnea compartment ($V_{n_{sigh}}=V_{n_{eupnea}}=-90\;\text{mV}$), the period of sighs decreased sharply with a *V*_K_ increase (Fig. 8*B*, green curve). When *I_h_* half activation in sigh compartment became more depolarized (Fig. 8*A*, red curve), the sigh period still responded to increase in *V*_K_, but this dependence was less steep (Fig. 8*B*, red curve). When the activation curve in sigh compartment was moved even further to the right (*V_n_* = −70 mV; Fig. 8*A*, black curve), the sigh compartment became insensitive to increase in *V*_K_ (Fig. 8*B*, black curve). In contrast, changes in *I_h_* activation threshold did not affect the period of eupnea oscillations (Fig. 8*C*). Thus, our model predicted that the differential sensitivity to overall excitability between the sigh and eupnea might be linked to distinct activation properties of the *I_h_* current in the two subpopulations.

## Discussion

In this work, we combined experimental and modeling approaches to test the hypothesis that sigh and eupnea are generated by different subpopulations of neurons in the embryonic preBötC network. We developed a two-compartment mathematical model of the respiratory network with eupnea and sigh subpopulations being identical except for three parameters. In the model, we assumed that the activity of a persistent sodium current drives fast oscillations in the eupnea compartment while the release of Ca^2+^ from intracellular stores generates slow oscillations in the sigh compartment. We established that only the combination of an inhibitory connection from the eupnea to sigh compartments and a reciprocal excitatory connection correctly reproduced the *in vitro* experimental recordings. Our model also predicted that sighs are less sensitive than eupnea to changes in [K^+^]*_o_* concentration; this prediction was confirmed experimentally. Finally, we found that the hyperpolarization-activated current, *I_h_*, had to be present in both sigh and eupnea subpopulations in order to produce typical biphasic sigh bursts. Another prediction of our model is that *I_h_* had a higher activation threshold in the sigh subpopulation, suggesting a stronger activation at physiological resting membrane potentials. Overall, our combined *in vitro* and *in silico* findings support the conclusion that sigh and eupnea were generated by two distinct subpopulations and provided the basis for continuing to understand the cellular and network mechanisms by which the sigh rhythm is produced.

It has been previously shown that the respiratory network of the preBötC starts to generate sighs at embryonic ages (Chapuis et al., 2014). We mainly used these recent data to build our model, and therefore also performed our *in vitro* experiments on preparations obtained at embryonic stages. However, the preBötC continues to generate sighs *in vitro* later in development. It is very likely that changes/maturation/adaptation of the mechanisms underlying sigh generation occur at later developmental stages. For example, it is known that chloride-mediated signaling undergoes significant functional changes during the perinatal period. Because it has been shown that inhibition plays an important role in coordinating the two components of a sigh burst, maturation of glycine signaling might be one of the parameters undergoing developmental changes with potential developmental consequences on sigh rhythmogenesis. In rat, Ren and colleagues (2006) have shown that the transition from excitatory to inhibitory effects of chloride-mediated conductances occurs right prior birth. Here in the mouse, experiments were performed at the corresponding time (the day directly preceding birth), suggesting that chloride-mediated events might already be inhibitory. However, Koch and colleagues (2013) recently showed that sigh generation requires the maturation of some membrane and synaptic properties. Performing physiological experiments at postnatal stages would be informative in testing the relevance of our findings at more mature states.

Although our present results are consistent with a two-subpopulation mechanism underlying fictive eupnea and sigh generation, the alternative hypothesis that both rhythms are generated by a single network cannot be completely excluded. Indeed, if we increase the ER Ca^2+^ storage in the eupnea compartment (parameter λ), the model can produce both large amplitude Ca^2+^ oscillations and fast *I*_Na_*_P_* oscillations within the same compartment. Such a mechanism has been reported previously (Jasinski et al., 2013). However, the voltage profile of such single-compartment model is missing several crucial aspects of *in vitro* sigh recordings. First, the high value of *g*_Na_*_P_* needed for Ca^2+^ oscillations reduces the period of sigh-like oscillations to the order of seconds instead of minutes. We found it is impossible to produce minute-long Ca^2+^ oscillations in this configuration: the excessive depolarization provided by the large value of *g*_Na_*_P_* activates *I*_Ca_ too fast and triggers Ca^2+^ release from the ER a few times per second. Second, we were unable to reproduce the change in the sigh shape from biphasic to monophasic after blocking inhibitory synaptic connectivity in a single-compartment model. And finally, as reported previously (Jasinski et al., 2013), this latter model is able to generate sigh in a stable manner only within a very narrow parameter regime. Altogether, these observations favor the hypothesis of two subpopulations involved in the generation of the two different types of inspiratory activity. Another possibility for sigh/eupnea generation is the involvement of complex network connections among preBötC cells. Indeed it has been shown that network connectivity can lead to self-organized activity on different time scales (Eytan and Marom, 2006; Bullmore and Sporns, 2009). In addition, the existence of specific metabotropic connections that distinguish sighs from eupneic activity has been proposed (Lieske and Ramirez, 2006b). However, in the absence of clear experimental evidence on connectivity among the cells in preBötC, we have not made any explicit assumptions on the connectivity within the network.

Although including a number of essential ionic channels and cellular pathways, our model remains simplistic and can only reproduce some of the features of respiratory activity generated by the preBötC network. For instance, although our model displays a prolonged period of inactivity after each sigh, it is still much shorter than the post-sigh apnea observed in our electrophysiological recordings. It is thought that the post-sigh pause *in vivo* is associated with chemomodulation and/or with Hering−Breuer reflex inhibition of inspiration via vagal stimulation of stretch receptors. However, because this short pause is also observed in reduced preparations, such as transverse brainstem slice and even island preparations, it is also likely to derive from specific characteristics of the intrinsic membrane properties of inspiratory neurons (Lieske et al., 2000; Tryba et al., 2008; Viemari et al., 2013; Chapuis et al., 2014). Indeed, intracellular recordings have shown that during sighs, inspiratory neurons are activated to depolarized levels that are higher than those occurring during eupneic bursts. This stronger activation of bursting mechanisms thus results in shifting the membrane potential further from triggering thresholds, leading to an increased refractory period that causes the subsequent eupneic burst to be delayed. It is also possible that some mechanisms essential for post-sigh apnea are not explicitly modeled in our simulations and are required to more accurately reproduce this feature of sigh activity. Another result of model simplification is that partial blockade of Ca^2+^ conductance decreased eupnea frequency in our model, while the slice recordings showed instead an increase. Again, this is probably due to an incomplete list of ionic currents incorporated into our model. One possibility is that Ca^2+^-activated K^+^ channels could lead to an overall hyperpolarization and decrease in eupnea frequency. It is also very likely that applying cadmium *in vitro* does not only affect calcium-dependent conductances in the slice but might also cause changes in other calcium-dependent mechanisms, including in synaptic interactions (Lieske and Ramirez, 2006a; Koch et al., 2013), and is therefore not as specific as changing a single conductance as we were able to do in the model. Third, another difference of the model is that reducing the synaptic inhibitory conductance to zero does not induce an increase in eupneic burst amplitude as observed experimentally. This can be explained by the fact that the synaptic wiring between the two compartments in our model is thus far very simplistic. Indeed, our present data and previously published studies (Winter et al., 2009; Morgado-Valle et al., 2010) suggest that inspiratory neurons receive inhibitory inputs during eupneic bursts that participate in limiting their burst size. In addition, it has to be noted that our data assigned a new functional role to glycinergic neurons within the preBötC network in the coordination of eupnea and sigh rhythms. Fourth, discrepancies also exist regarding the effect of blocking *I*_Na_*_P_*. While applying riluzole *in vitro* (at concentrations currently used by others) prevents both eupnea and sigh activities to be generated at comparable concentrations (a feature that might be specific to embryonic ages), blocking the persistent sodium current *in silico* leads first to a complete blockade of eupnea activity while sighs are still generated but with a smaller amplitude. Once again, this outcome probably reflected an incomplete set of membrane conductances in the different compartments of our model. Finally, it is possible that individual cell properties are more important for sigh generation than our model has assumed. A study by Tryba et al. (2008) indicated that there are some preBötC neurons that generate two different types of bursts at two different frequencies under the conditions of pharmacological blockade of synaptic transmission. These experimental findings suggested that a single preBötC neuron can generate both eupnea and sigh rhythms. However the same study demonstrated that such neurons are extremely rare (less than 1% of experimental cells), which make it highly unlikely that they generate the sigh burst in the network.

The present study also explored the potential role of *I_h_* in sigh generation. Our patch-clamp recordings performed on embryonic inspiratory neurons revealed that *I_h_* was expressed in a high proportion (∼86%) of the neuronal population sampled here. This was corroborated by the finding that *I_h_* must be expressed in both compartments of our model (and would therefore be expressed in the majority of network constitutive elements). However several previous studies performed in newborn rodents found that around half of the rhythmically active pre-Bötzinger interneurons are devoid of *I_h_* (Rekling et al., 1996; Thoby-Brisson et al., 2000; Picardo et al., 2013). This experimental disagreement might be due to the fact that our experiments were conducted at earlier developmental stage. This suggests that the prevalence for *I_h_* might change during development, being highly expressed at more immature states. Such difference also has functional consequences: *I_h_* blockade induces opposite effects on eupneic frequency in the embryo versus newborn, triggering a frequency decrease at postnatal stages and an increase before birth. Despite the fact that our data clearly show that *I_h_* activation is required for sigh generation in the embryo, it remains possible that this specific role also evolves during later stages of development. Testing this hypothesis would require additional experiment, which is beyond the scope of the study.

Another very important characteristic to be considered is intra-network synaptic connectivity. One primary limitation of our model is that it simulates cell subpopulations and thus ignores individual cell-to-cell interactions. Although we have not modeled explicitly the connections within each subpopulation, we assumed that excitatory synapses among cells are widespread and that blocking these connections would inevitably result in the absence of rhythmic output in our model. Second, our combined *in vitro* and *in silico* approaches point to a role of synaptic inhibition in sigh generation. The possible explanations for the monophasic shape of the sigh during strychnine application could be the absence of phase synchronization between sigh and eupnea oscillations, as shown in the newborn (Lieske et al., 2000) and in the embryo (Chapuis et al., 2014) or, in contrast, a complete synchronization of the sigh’s two components. The first depolarizing phase of the sigh is a presumed eupnea, which is followed by Ca^2+^ release from the ER. Such a mechanism would in turn require a precise balance between excitatory and inhibitory synapses. Thus, our model predicted that a mild decrease in synaptic inhibition would extend the time between the two phases of sigh events, thus resulting in separate monophasic bursts. But our model also predicts that a mild increase in synaptic inhibition would lead to a tighter synchronization and loss of the sigh’s biphasic shape. A full synchronization is theoretically possible, but we have no experimental evidence supporting this in the embryo.

The initial indication for the possible existence of two subpopulations involved in sigh and eupnea generation was the report of so-called “sigh-only” neurons in two separate studies (Tryba et al., 2008; Chapuis et al., 2014). These neurons, active exclusively during sigh events, were identified either by individual patch-clamp recordings or through calcium imaging, and in each case they constituted a very small population (suggested to be <5% of the neurons active during respiration-related activities). This population size imbalance was not directly taken into account in our model: the two compartments represented subpopulation entities without simulation of individual constituent elements. Consequently, we do not know how many cells are required to mimic the output of each subpopulation. However, the fact that we can fit our data only when the connection from the eupnea to sigh compartments is stronger than the reciprocal one is consistent with the assumption that eupnea subpopulation is much larger than sigh subpopulation. It is also possible that the two subpopulations have different morphologies. As previously shown, adding a dendritic compartment to a model of persistent sodium pacemaker enables the generation of slow Ca^2+^ oscillations (Toporikova and Butera, 2011). Thus, the sigh-only cells might have a larger dendritic tree that enables Ca^2+^ accumulation and slow calcium-dependent intracellular processes. Testing this possibility would obviously require additional experiments and simulations.

One important question is: could a small neuronal population cause synchronization among all neurons of the network? Inspiratory neurons voltage-clamp recordings previously published (Chapuis et al., 2014) and some of our current-clamp recordings (unpublished data) show that during a sigh burst, both discharge frequency of inspiratory neurons and synaptic drive potentials are more intense. It is impossible to determine which one is the consequence of the other, but it illustrates the fact that all neurons of the pre-Bötzinger network are strongly and probably reciprocally connected through glutamatergic synapses (Feldman and Del Negro, 2006). Therefore, it could be reasonable to consider that even if the sigh-only neuron population might be limited, it could significantly influence the whole network through an efficient recurrent excitatory loop targeting a large proportion of the constitutive neuronal elements of the network.

To our knowledge, our model is the first to accurately reproduce the bimodal discharge pattern of the preBötC network generating simultaneously, but with very different frequencies, eupnea and sigh rhythmic burst activities. Although our findings did not completely resolve the question of the mechanism of distinct rhythmic breathing activities, our results definitively support the hypothesis that sighs and eupnea are generated by separate networks within the preBötC. By continuing to combine physiological data with computational simulations, we will gain additional insights on mechanisms underlying sigh generation.

## Synthesis

The decision was a result of the Reviewing Editor Douglas Bayliss and the peer reviewers coming together and discussing their recommendations until a consensus was reached. A fact-based synthesis statement explaining their decision and outlining what is needed to prepare a revision is listed below. The following reviewers agreed to reveal their identity: Jan-Marino Ramirez and Jeffrey Smith

Both reviewers were quite positive about the work and the advance it will provide for the field. However, as you will note from their reviews (see below), there are a number of points that should be presented with greater clarity or better perspective. Of particular concern is a more careful discussion of the evidence for and meaning of the two neuron populations that are envisaged (e.g., do these represent different oscillators? different neurons?), how those are conceptualized within the model (e.g., how equate non-spiking compartments to "population" activity?), and alternative possibilities. These points can be handled largely by revising the text according to the reviewer's suggestions.

There was one point where additional experimentation was recommended - specifically, with respect to testing the central role for intracellular calcium release that was proposed. This could be tested in the slice preparation with appropriate and readily available pharmacological tools that disrupt calcium storage or release. If possible, it was felt that this suggested experiment would significantly enhance the paper.

We look forward to receiving your revision of the manuscript.

### Reviewer 1

This study combines computational and electrophysiological approaches to study the mechanisms underlying the generation of eupneic and sigh activity in the embryonic respiratory network. The study is of general interest because it addresses a fundamental question - how one network can generate two rhythms with very different physiological and pharmacological properties. The study is well analyzed and well written. However, I have several comments that need to be addressed.

### Major comments

(1) A major caveat is the fact that this study was performed in the embryonic network (E18.5, line 145). While the authors carefully discuss these caveats there are additional implications that need to be addressed. One major caveat is that chloride conductances are typically depolarizing early in development. If this is the case in the embryonic respiratory network - this would probably totally change the proposed model. This needs to be emphasized - e.g. on line 494. Another example: the authors find that the eupneic rhythm stops at 3mM potassium, which could be specific for embryos. It is known that the rhythm of neonates stays on at 3mM.
(2) Along the same lines, the authors do not include the ICAN in their model, and in their experiments. To some authors this is a critical current. I am personally fine with restricting the model to INap, but this caveat should be emphasized more. Perhaps one explanation and rationale is the fact that the authors performed these experiments in the embryo where they find that riluzole blocks the eupneic rhythm (line 347). The eupneic network in neonatal and more mature preparations is not blocked by riluzole alone - as it also requires FFA (blocking ICAN) to fully block eupneic respiratory network activity. Also, there is evidence that ICAN matures (e.g. Pena et al. 2004 suggested this). This could be added to the discussion. Along the same line, line 562 - not everyone one shows that riluzole completely blocks eupnea - this could be an embryonic speciality...
(3) The authors assume that the sigh is based on slow calcium oscillations (e.g. line 236). Specifically they propose that calcium is released from the ER. The authors further hypothesize a slow inactivation of IP3R. While the authors tested the role of voltage dependent calcium currents and the Ih current, they did not experimentally test the possibility that calcium is released intracellularly. To me this is a real caveat. The lack of experimental evidence for the core mechanisms needs to be addressed in the discussion, or ideally in experiments.
(4) The authors propose two populations: a sigh and eupneic population (e.g. line 262). Do they think that these are really two separate populations? I am not sure that there is real experimental evidence for this. After all, the "sigh only" neurons are extremely rare. We know that there are distinct synaptic mechanisms, but this doesn't necessarily mean that there are distinct populations of neurons. As for the distinct synaptic mechanisms, the authors should cite the second Lieske and Ramirez publication in 2006, in which these authors characterize the specific metabotropic connections that distinguish sighs from eupneic activity. This to me seems very relevant here. One potential place to add this discussion is line 522, as there is real experimental evidence that there are differences in connectivity. Along the same lines: on line 547 the authors state that calcium mechanisms could be involved in synaptic mechanisms. There is clear evidence for this: e.g. the studies that show that the PQ type calcium current contributes to the glutamatergic synaptic transmission (Koch paper, Lieske paper).
(5) One key argument for their model is the experimental finding that strychnine renders the sigh rhythm mono-phasic (lines 292, 303 etc). The authors cite the Lieske (2000) study. However, the Lieske study showed that strychnine abolishes the phase-locking. The sigh now comes randomly, and is not locked to the eupneic rhythm, i.e. that's why the sigh-burst becomes mono-phasic, not because it is now totally synchronized with the eupneic burst. This needs to be addressed. The authors discuss this on line 597 - but they don't explicitly state that the experimental evidence does not NOT suggest a complete synchronization.
(6) Line 323: The authors repeated exactly the identical cadmium experiment that was published already by Lieske et al 2000. This should be cited appropriately and mentioned. Lieske, Koch and coworkers did not just do the P/Q type experiments.
(7) Line 72: the authors state <5% of inspiratory neurons are "sigh-only cells". Unfortunately, we don't know how many of the cells are sigh only - it could be less or more. So state: "the existing recordings suggest that these neurons constitute less than 5% of the inspiratory neurons". But, the critical question that needs to be discussed in order to support the model is the following: could such small neuron population cause synchronization among all neurons? Are the authors implying that this is THE subpopulation that their model is based on?

### Specific Comments

(1) line 473: Please state "in the preBötC of the embryonic network".
(2) Line 67: The authors state " all neurons active..." but this is not the case - and indeed the entire study hinges on the fact that not all neurons... In any case, change to "the majority"...
(3) Line 94. It would be better to use here the word compartment instead of subpopulation leading to the assumption that you consider neuronal networks and not a two compartment model.
(4) Line 100. I would recommend to stay consistent with the terminology, since all currents except the leak current and CAN current are voltage-gated, for example voltage-gated persistent sodium current, voltage-gated Ih current and etc, not just voltage-gated calcium current leading to the assumption that it's the only voltage-gated current.
(5) Line 103. Ik is missing from the equation (1)
(6) Line 104. Ica current is described similar to non-inactivating persistent sodium current, except the reversal potential is very high for Ca currents. Its 150mV. Please, explain in the text why you introduce Ca2+ current as none -inactivating, and not as m2h and why the reversal potential needs to be so high. Does it change the dynamics of the model if you set Vca close to the experimental value?
(7) Line 104-105. Ih current. The authors describe this current as Ih=ghninf(V-Vh) and refer to ninf as activation variable. According to the parameter values this is an inactivation variable, please change the description. The authors should explain why an activation variable was not introduced to describe the Ih current. According to the figure 7A, the reversal potential is about -60mV, but the author used +30 mV, please mention this in the text and explain the necessity of using this value.
(8) Line 107. The authors should define ksyn parameter in the text.
(9) Line 108. Formula (9). Following the text description, hinf should be written as hinf(V)
(10) Line 113. The authors should define sx in the text.
(11) Line 145-146. "We performed all physiological experiments..." this sentence is not clear.
(12) Table 1: What are the parameter's units?

### Reviewer 2

In this combined in vitro experimental and computational modeling study the authors test their hypothesis that the inspiratory network in the embryonic rodent (mouse) preBötzinger complex (preBötC) network generates two distinct, established patterns of rhythmic inspiratory network activity-"eupneic" and sigh bursts- by separate interacting neural components involving distinct biophysical mechanisms. Sighs are thought to be physiologically important and appear to emerge prenatally in late embryonic stages as the authors' data emphasize. Their proposed model consists of two synaptically coupled distinct "compartments" to represent conceptually the two neuronal sub-populations responsible for the rhythmic eupneic and sigh burst generating biophysical processes. This model appears to qualitatively capture a number of experimental observations presented in this paper from electrophysiological measurements in rhythmic embryonic mouse in vitro slice preparations. This correspondence, which generally supports their hypothesis, includes importantly a possible mechanistic explanation for the different time scales of the generation of these two forms of inspiratory activity, dependence of the sigh biphasic burst shape on inhibitory coupling from the eupneic compartment as well as the involvement of Ih, the dependence of sigh burst generation on calcium-based mechanisms, and the dependence of eupneic and sigh burst generation on persistent sodium current. The Discussion of limitations of the model/model-data discrepancies resulting from simplifications, assumptions, and parameter choices, and the fact that the model cannot definitively exclude that the calcium- and sodium-based mechanisms co-exist in the same neuronal population, is appropriate.

The novel results presented in this study provide new insights into the possible biophysical processes generating these two behaviorally important forms of circuit activity. Another value of this generally well-executed exploratory study is that it attempts to synthesize existing and new data-model based observations in a framework that can guide more extended analyses of the fundamental mechanisms involved.

I have several suggestions for revision.

p. 6, para. 1, last sentence. "Each compartment of the model represents a cluster of such preBötC neurons that are interconnected with excitatory synaptic coupling." This statement is not strictly true because each compartment cannot represent all of the dynamic behaviors that an all-to-all excitatory-coupled homogeneous population of neurons can. Furthermore, readers may be confused that the eupnea "population" makes an inhibitory connection with the sigh "population". Thus I do not think that it is appropriate to refer in the text to their single-compartment eupnea and sigh models as representing "populations" or "networks".

p. 14, para. 2. "Each compartment of the model represents a neuronal group of preBötC neurons connected through excitatory synapses but without any specific pacemaker properties." The meaning of this statement is unclear. Each compartment can oscillate intrinsically, which constitutes a pacemaker property. Please revise to clarify the intended meaning.

p. 24, para. 2. To provide the reader with insight into the relevant dynamics, it would be useful to explain why the sigh burst exhibits a biphasic shape only when Ih is present in both model compartments.

p. 25, para. 1. What is the Ih half-activation voltage from the authors' measurements, and how well does this fit with the model predictions for the voltage that yields the most appropriate behavior of the model.

p. 28, para. 1, last sentence. "...we assumed the all-to-all topology to reduce the number of assumptions." No such assumption is made explicitly in the model because networks are not modeled here.

p. 29, second to last sentence. "...this synaptic signal should be implemented in the model of individual neurons rather than population of neurons." The meaning of this sentence is unclear since the authors have implemented a "model of single neurons". Please clarify.

p. 31, para. 2. "In fact each compartment of the model represented a network of cells that are homogeneously coupled to each other". Refer to comment p. 6, para. 1 above.

Figures and Legends.

Fig. 1 legend. "...except for ER capacity, INaP, and Ih, the sign and eupnea models are identical." Insert "parameter values for" before ER capacity.

Figure 6. Give the value of Vk used in the model to correspond to 4 mM extracellular K+ as labeled. The image shown in part B is too small to see the labeled neurons of interest. An enlarged image should be shown.
